# SET-bullying: presentation of a collaborative project and discussion of its internal and external validity

**DOI:** 10.1186/s13104-016-2014-6

**Published:** 2016-04-12

**Authors:** Alexandros-Georgios Chalamandaris, Michèle Wilmet-Dramaix, Mike Eslea, Sigrun Karin Ertesvåg, Danielle Piette

**Affiliations:** School of Public Health, Université Libre de Bruxelles (ULB), Route de Lennik 808 CP596, 1070 Brussels, Belgium; School of Psychology, University of Central Lancashire, Darwin Building, Preston, Lancashire PR1 2HE UK; Norwegian Centre for Learning Environment and Behavioural Research in Education, University of Stavanger, 4036 Stavanger, Norway

**Keywords:** School-based anti-bullying interventions, Internal validity, External validity, Effectiveness, Time, Project description

## Abstract

**Background:**

Since the early 1980s, several school based anti-bullying interventions (SBABI) have been implemented and evaluated in different countries. Some meta-analyses have also drawn conclusions on the effectiveness of SBABIs. However, the relationship between time and effectiveness of SBABIs has not been fully studied. For this aim, a collaborative project, *SET*-*Bullying*, is established by researchers from Greece, Belgium, Norway and United Kingdom. Its primary objective is to further understand and statistically model the relationship between the time and the sustainability of the effectiveness of SBABI. The secondary objective of *SET*-*Bullying* is to assess the possibility of predicting the medium-term or long-term effectiveness using as key information the prior measurement and the short-term effectiveness of the intervention.

**Results:**

Researchers and owners of potentially eligible databases were asked to participate in this effort. Two studies have contributed data for the purpose of *SET*-*Bullying*. This paper summarizes the main characteristics of the participating studies and provides a high level overview of the collaborative project. It also discusses on the extent to which both study and project characteristics may pose threats to the expected internal and external validity of the potential outcomes of the project.

**Discussion:**

Despite these threats, this work represents the first effort to understand the impact of time on the observed effectiveness of SBABIs and assess its predictability, which would allow for better planning, implementation and evaluation of SBABIs.

## Background

Since the pioneering anti-bullying intervention in Norway in the early 1980s (as described by Roland citing others [1]), several other anti-bullying interventions have also been implemented in different countries. In addition, some meta-analyses [2–5] have been conducted in order to draw conclusions on the effectiveness of School Based Anti-Bullying Interventions (SBABIs).

Despite the work done so far in the field of anti-bullying research, the relationship between the time and the effectiveness of anti-bullying interventions has not been fully studied. Identifying this gap in the literature, Evers et al. [6] wonder “*whether shorterduration of evaluations helps or hurts a study*”.

In a meta-analysis on the effectiveness of SBABIs, Ttofi and Farrington found that SBABIs with longer duration seem to be more effective than shorter SBABIs, and they note that “*it could be that a considerable time period is needed inorder to built up*” the effectiveness of a SBABI [5]. However, the pattern of this “*built up*” and the evolution of SBABI effectiveness over time has not been explored.

In order to address this, a collaborative project was established in 2009, involving researchers from Greece, Belgium, Norway and United Kingdom. More precisely, a Greek (AGC) and two Belgian (MWD and DP) researchers from Université Libre de Bruxelles have contacted several researchers, based on the process described below. SKE from Norway and ME from United Kingdom, whose studies met the inclusion criteria described below, agreed to participate in this collaborative project.

The aim of this collaborative project is to explore the form and the magnitude of the relationship between time and the effectiveness of SBABIs. Its name is *SET*-*bullying*, an acronym standing for statistical modelling of the effectiveness of school based anti-bullying interventions and time.

This paper is a project note aiming to describe *SET*-*Bullying* and the characteristics of the studies that have contributed data. It also discusses the extent to which both study and project characteristics may pose threats to the internal and external validity of the potential outcomes of the project. The details on the analysis methodology and the corresponding results will be described elsewhere.

## Research hypothesis of *SET*-*bullying*

The effectiveness of SBABIs is concluded based on changes in bullying-related outcomes (BRO). The BROs usually include measures of pupil self-reported frequencies of bullying, opinions regarding bullying, reports of behaviours or intentions of behaviours against bullying incidents [7]. This information is collected through reports of pupils, school personnel, teachers or other stakeholders [7].

Prior to the implementation of a SBABI, the BROs reflect a certain status of bullying in the school environment. The implementation of the SBABI intends to alter the status of bullying and this alteration is expected to be reflected with respective modifications in BRO variables. The direction and the magnitude of this alteration, measured as the difference in BROs values before and after the SBABI, is used to derive conclusions on the effectiveness of the SBABI.

It is not clear how this alteration is produced over time. It could follow a pattern of a gradual built up of the effectiveness over the course of the SBABI, reaching a pick point close to the end of the SBABI. Other potential patterns may include a fast built up of the effectiveness and then reaching a plateau until the end of the SBABI, or a peak point after the end of the intervention as some incubation period may be needed for the maximum effect to appear. The latter assumption is also suggested by Ttofi and Farrington [5]. Additionally, it may be that the pattern is not monotone.

Independently of the pattern of the assumed built up until the end of the SBABI implementation, it is assumed that any change in terms of effectiveness will gradually fade out over the course of time after the end of the intervention, as the school community returns to its everyday routine.

The extent and the speed of both the assumed built up and fade-out are dependent on the SBABI characteristics. A more effective SBABI may produce both a more intense built up and more sustainable effect after its end, as opposed to a less effective SBABI.

*SET*-*Bullying* hypothesises that the afore mentioned relationship between the time and the effectiveness of a SBABI can be described and statistically modelled, independently of its pattern or magnitude. It also hypothesises that the form of the relationship may be similar for outcomes measuring the same concepts, even if different instruments have been used for different SBABIs. The numerical expression of this relationship is expected to fluctuate as a result of the SBABI characteristics and effectiveness. The knowledge of the form of this relationship would provide insights helping to more efficiently design and execute studies to evaluate the effectiveness of SBABI.

Additionally, if the statistical modelling reveals a consistent pattern of the investigated relationship for several BROs, then it could be also used for projections of health promotion needs, with respect to bullying, on a medium-term or long-term basis. Therefore it would provide useful insights for long-term planning of anti-bullying strategies to the population to which the SBABI is implemented. Thus, it would allow policy-makers to leverage more efficiently the available resources.

## Objectives of *SET*-*bullying*

In order to evaluate the afore mentioned research hypothesis, the primary objective of *SET*-*Bullying* is to further understand and statistically model the relationship between the time and the sustainability of the effectiveness of SBABI. This will be assessed based on pupils’ self-reported frequencies of been bullied and bullying others.

The secondary objective of *SET*-*Bullying* will aim to assess the possibility of predicting the medium-term or long-term effectiveness, in terms of self-reported frequencies of been bullied and bullying others, using as key information the prior to the intervention measurement and the short-term effectiveness (i.e. the first post-intervention measurement).

## Identification of the participating studies

Chalamandaris and Piette [7] have conducted a literature review including 62 articles from peer-reviewed journals, which “*present[ed] information on the evaluation design used to assess the effectiveness of*” SBABIs [7]. These 62 articles were published *“prior to the end of January 2008”* [7] and they formed the basis for this work. Based on the articles included in this review, 27 articles, corresponding to 22 unique studies, were identified as satisfying the following eligibility criteria:

- Containing data on the evaluation of the effectiveness of SBABI.
- Having at least one measurement prior to the intervention and two data collections at different time points during or post the SBABI.
- Providing the possibility to identify measurements of the same group/cluster of pupils (i.e. pupils or classrooms or grades or schools) over time.

The next step was to contact and inform the corresponding authors, for which correspondence information was retrieved, regarding *SET*-*Bullying* and asking about their willingness to collaborate in this project.

In total, three research teams responded positively and provided their database. Out of these three databases, two could be used for analysis. These refer to study *DFE*-*SHEFFIELD* from United Kingdom [8] and study *RESPEKT* from Norway [9]. Thus, only two of the initially identified 22 studies are included in *SET*-*bullying*.

## Description of the participating studies

The following subsections present and compare the main characteristics of the studies *DFE*-*SHEFFIELD* and *RESPEKT* with respect to the type of anti-bullying intervention and the research design that are of interest for the purposes of *SET*-*bullying*. More information about the theoretical context and the study characteristics of each of these interventions can be found in the original articles [8, 9].

### Description of the anti-bullying interventions

*DFE*-*SHEFFIELD* was designed to target bullying behaviour [8], while *RESPEKT* addressed bullying together with “*disobedience*” and “*general off-task behaviour*” of pupils [9]. Both interventions were implemented by the school personnel. The research team of each SBABI provided training and support to the school personnel with regards to the implementation. *RESPEKT* aimed to enhance the “*classroom leadership*” of teachers [9]. This way, teachers would act as an “*authority*” [9] in the classroom putting in place rules of expected and allowed behaviours. On the other hand, *DFE*-*SHEFFIELD* used a different approach by implementing a “*whole-school*” approach [8], which included help to individual pupils and prevention of bullying in the playground through environmental changes and curricular activities. *RESPEKT* also implemented a “*whole school approach*” [9] with activities targeting the “*individual, classroom and school levels*” [9].

### Study design characteristics

Table 1 presents a summary of the key study design characteristics of the two studies. Regarding the number of pupils appearing in Table 1, it should be noted that the number of pupils that participated in the questionnaire administrations may not always correspond to the number of pupils that have participated in the SBABI. In *RESPEKT*, the SBABI was implemented in all grades of primary schools but only the three older grades provided data for evaluation [9]. None of the two studies included any control or comparison group [8, 9]. Study *DFE*-*SHEFFIELD* was designed to compare changes from baseline [8], while study *RESPEKT* was designed to compare “*adjacent cohorts*” [9].

**Table 1:** Description of studies included in *SET*-*Bullying*

Database name	
RESPEKT [9]	DFE-SHEFFIELD [8]
Schedule of study measurements^a^	
*T1*: Baseline, May 2002, −3 months *T2*: End of intervention, May 2003, 9 months *T3*: Follow-up 1, May 2004, 21 months *T4*: Follow-up 2, May 2005, 33 months	*T1*: Baseline, late November/early December 1990, −9 months *T2*: End of intervention, late November/early December 1992, 15 months *T3*: Follow-up, late November/early December 1993, 27 months
School grades (age in years) of pupils	
Primary school: 5–7th (age: 11–13)	Primary school: 3–6th (age: 7–11)
Secondary school: 8–10th (age: 14–16)	Secondary school: 7–18th (age: 12–18)
Number and type of study groups/clusters	
18 primary school grade groups	17 primary school groups
6 secondary school grade groups	7 secondary school groups
Number of pupils at each study measurement^b^	
Primary school: *T1*: 417, *T2*: 414, *T3*: 413, *T4*: 414	Primary school: *T1*: 2617, *T2*: 2481, *T3*: 655
Secondary school: *T1*: 329, *T2*: 354, *T3*: 364, *T4*: 365	Secondary school: *T1*: 4123, *T2*: 4624
Number (percentage) of female pupils in each measurement^b^	
Primary school: *T1*: 228 (54.7 %), *T2*: 222 (53.6 %), *T3*: 199 (48.2 %), *T4*: 210 (50.7 %)	Primary school: *T1*: 1350 (51.6 %), *T2*: 1234 (49.7 %), *T3*: 317 (48.4 %)
Secondary school: *T1*: 178 (54.1 %), *T2*: 194 (54.8 %), *T3*: 166 (45.6 %), *T4*: 170 (46.6 %)	Secondary school: *T1*: 1977 (48.0 %), T2: 2272 (49.1 %)

^a^ Time in months corresponds to time relative to the start of the intervention

^b^ Number of pupils includes all pupils with a least one non-missing value in any of study outcomes

Both studies included questionnaire administrations to pupils yearly or bi-yearly. For each study, the period of pupil questionnaire administration was kept the same, in order to control for any seasonality effect [9]. Study *DFE*-*SHEFFIELD* included three questionnaire administrations to pupils [8], while study *RESPEKT* included four [9]. Nevertheless, there is a 3 years distance between the first and the last questionnaire administrations for both studies. Table 1 presents the number of the study measurements in pupils, their timing as well as their time distance in months from the start of each intervention. In *RESPEKT*, questionnaires were administered by classroom teachers [9] while in *DFE*-*SHEFFIELD* by a different teacher than the classroom teacher [8].

## Bullying-related outcomes for *SET*-*bullying*

Both studies have included data collection from pupils and school personnel. Each study has included measures of different concepts, relevant to the SBABI objectives.

For the purpose of *SET*-*bullying*, only the data from questionnaires to pupils, referring to self-reported frequencies of been bullied and bullying others, were selected for further analysis. These outcomes are directly related to the primary objectives of the SBABIs, therefore they should reflect the effectiveness of the SBABIs. Using pupils self-reports has some advantages [7], since pupils, being the protagonists, are the ones to assess if an incident qualifies as bullying or “*friendly forms of teasing*” [10]. At the same time these were the only BROs that were assessed by both studies, thus giving the opportunity to explore the objectives of *SET*-*Bullying* in both databases.

In both studies, the data collection was based on questionnaire items referring to specific bullying behaviours as well as items on general bullying behaviour [8, 9]. For *RESPEKT* these items were summarized in respective scales and the information is available in both formats (i.e. scale scores and individual scale items) [9], while in *DFE*-*SHEFFIELD* results were reported for each item separately [8].

The recall time for pupils to report whether they were bullied or they bullied others differed from five school days or to the last school term for *DFE*-*SHEFFIELD* [8] or the school year for *RESPEKT* [9].

Regarding BROs from other informants, study *RESPEKT* collected information from teachers only in the first two study measurements [9]. Since the aim of *SET*-*Bullying* was to model the relationship between bullying related outcomes and time over several measurements across time, these data could not be used for analysis. In study *DFE*-*SHEFFIELD*, there was only qualitative information that was collected from head-teachers [8], which could not be used neither for the aims of *SET*-*bullying*.

## Analysis methodology used in *SET*-*bullying*

In terms of analysis, the first challenge lies on both studies having used different instruments for data collection. Therefore, we will explore methods of harmonising the format of the outcomes of *SET*-*bullying*, such as principal components analysis [11, 12]. Such a harmonisation will facilitate the implementation of the same analysis methodology in both databases. Despite having used different instruments, it is assumed that both studies have measured the same outcomes. Therefore, we assume that the relationship between time and effectiveness, as reflected in these outcomes, will be observed in both studies, on a common ground, independently of a the instruments used.

Two subsequent challenges for analysis are linked to the particularities of the structure of the data of the two studies participating in *SET*-*Bullying*. It is expected that the reports of students within the same group/cluster (classroom or school grade or school) may be more correlated as opposed to reports of students from different classrooms or schools (i.e. hierarchical structure of the data). Also, measurements from the same pupils are expected to be correlated over time (i.e. longitudinal structure of the data). However, both studies have used anonymous questionnaires. Therefore, the responses of pupils can only be traced over time aggregated in groups/clusters, i.e. school grades for *RESPEKT* and schools for *DFE*-*SHEFFIELD*. The analysis methodology of *SET*-*Bullying* will take into account the hierarchical and the longitudinal structure of the data, using mixed effect model methodology [13]. The analysis will also try to explore differences in the form and magnitude of the relationship by pupils’ gender and age group. Therefore, these terms will be included in the statistical models.

## Discussion of internal and external validity

The purpose of this article is to describe a collaborative project named *SET*-*Bullying*, aiming to further understand and statistically model the relationship between effectiveness of SBABI and time, as well as, to assess its predictability.

This effort is a secondary analysis of data from SBABIs implemented in Norway [9] and United Kingdom [8]. Both studies were designed under different contexts, with different characteristics in order to conclude on the effectiveness of SBABIs [8, 9]. *SET*-*Bullying* performs a secondary analysis of the original available data in a different context and for a different purpose. It does not aim to reproduce what has been previously reported.

Any strengths or limitations of *SET*-*Bullying* are deriving from, in one hand, the strengths and limitations of the studies that have contributed data, and on the other hand, the strengths and limitations deriving from the methodology used in this collaborative project. In the following sections, the strengths and limitations are discussed using the criteria suggested by Windsor et al. [14] and Green and Glasgow [15], in terms of internal and external validity respectively. These criteria were previously discussed for bullying research by Chalamandaris and Piette [7].

### Internal validity

Windsor et al. [14] have proposed eight threats to the internal validity of study conclusions. Based on these criteria, any results from *SET*-*bullying* may be subject to bias due to:

1. Any kind of historical effect, which concomitantly to the SBABIs, could have modified pupils’ conceptualization of perception of bullying or could have influenced directly pupils’ behaviour, feelings, perceptions with respect to bullying [7, 14]. Although this kind of bias cannot be excluded in none of the studies, there is no evidence that such an event may have introduced it.
2. “*program or participant maturation*” [14]. Developmental changes in childhood and puberty may affect bullying related behaviours and its reporting independently of the implementation of a SBABI [7]. In parallel, the long-term implementation of a SBABI is expected to affect the cognitions of those directly or indirectly involved in it (i.e. pupils, teachers, school management and other key stakeholders) [7]. This could potentially lead to changes in the way that a SBABI is implemented in the school environment [7]. For both studies, the implementation of SBABI and the data collections expanded over several years.
  Any vulnerability on the conclusions due to this dual maturation effect is also impacting the results of *SET*-*bullying*. Statistical analysis is planned to account for changes in pupils’ age in the results. However, the observed pattern in the relationship between time and BROs may be confounded by such an effect. Nevertheless, stability over time regarding the SBABI or its participants, would be unrealistic in real-life contexts of bullying research.
3. Not honest or “*socially… desirable*” [14] responses. For pupils to admit in a questionnaire item that they bully others or that they are the victims may be rather challenging. Eslea and Smith [8] discuss this issue as a potential explanation of the apparent differences between the number of girls reporting bullying others and being bullied.
  In a study comparing the validity of self-reports versus peer nominations of bullying and victimization, Lee and Cornell [16] found greater disagreement between the two forms of reports of bullying as opposed to those of victimization. They suggest that perhaps “*it is easier for a student to recognizethat he or she is being bullied; a bully may not recognize that his or heraggressive behaviour constitutes bullying*” [16]. On the other hand, Eslea and Smith [8] discuss a similar argument, especially for indirect bullying, but at the same time they question if the differences in the results between boys and girls could be due either “*lack of awareness, or perhaps honesty*”.
  Regarding any differences in pupil responses based on the use of anonymous versus non-anonymous questionnaires, Lee and Cornell [16] discuss the results from other studies (Chan et al. 2005; O’Malley et al. 2000; van de Looij-Jansen et al. 2006) that have shown no evidence of differences in reporting of several types of behaviours, including bullying.
  Reporting having bullied others appears to be the most difficult or challenging behaviour for a pupil to report. This may be less of an issue for a pupil to report been bullied. In any case, for both outcomes, any difficulty in reporting constitutes a threat to the internal validity. None of the studies has sound evidence for such a bias was introduced. However, none of them can be considered as immune from this bias. This vulnerability is also carried forward to the collaborative project.
  A trend of pupil reports towards “*socially and programmatically desirable*” [14] responses cannot be excluded, especially for the measurements after the initiation of the SBABI. This can confound the relationship between time and effectiveness since the post-SBABI measurement may not fully reflect the SBABI effectiveness. This vulnerability can impact both the study and project results.
  In addition to voluntarily alteration of pupils’ reports, it is likely that pupils may modify their report due to the multiple measurements. Any sensitization effect, due to the repeated measurements, may be considered negligible since the shortest time distance between two consecutive measurements was 1 year. Therefore, it seems unlikely that pupils may have remembered their responses and therefore replicated them.
4. “*Instrumentation*” [14] and changes in the perception of questionnaire items by the pupils [7]. Eslea and Smith [8] discuss the impact on increased awareness of pupils in bullying incidence and suggest that this may resulted in increased reporting of minor bullying incidences that would not have been reported otherwise. This kind of bias cannot be excluded for *RESPEKT* as well.
  This type of bias influences the later study measurements and not the baseline measurements. Therefore, it may confound the relationship of effectiveness of SBABI with time and constitute a threat to the internal validity of *SET*-*Bullying* results.
  In parallel, pupils’ self-reports may vary depending on the period of the school year that bullying is measured. It is reasonable to assume that the level in the classroom or the pupil reported severity and frequency of bullying may be different if a measurement takes place in the middle of the school year or towards its start or its end, closer to the school vacation periods. By study design, *DFE*-*SHEFFIELD* and *RESPEKT* ensured that all measures take place at the same period in the school year, in order to minimize such a seasonality effect. Therefore, any such vulnerability is less likely to also affect the results of *SET*-*Bullying*.
  An additional point, related to the use of questionnaires in the assessment of the effectiveness of SBABI, is the difference in the recall time between studies. In each of the included studies, pupils were asked to report bullying incidents in the past. The recall time frame varied from five working days to last term [8] and last school year [9]. This variation between studies may explain some of the observed heterogeneity in their results.
  In relation to the instrumentation, Ryan and Smith [17], in their recommendations, suggest the collection of information through “*multiplemethods and multiple informants*” as well as the collection of “*qualitativedata*” in order to be able to assess the consistency in SBABI results and “*tocontextualize implementation and outcome data*”. Despite the fact both studies collected data in accordance with these recommendations, the collaborative project considers for analysis only pupils’ self-reports on frequencies of being bullied and bullying others. Given the afore mentioned limitations that are linked to pupil self-reports, the fact that *SET*-*Bullying* used only pupil self-reports of bullying others or been bullied is a threat to the internal validity of its results. Any attempt for contextualization of the results of *SET*-*Bullying* will be based on the input by the research teams owning the original databases.
5. The statistical methodology used in the analysis. Since the original data have been provided for analysis, any limitation due to the statistical analyses in the original studies does not impact the project results.
  However, it should be noted that *RESPEKT* was designed as an “*adjacentcohort design*” study [9]. This principle has guided the analytical methodology of the original study report [9]. In *SET*-*Bullying*, the same data will be analysed as repeated measurements using mixed effect models. Therefore, the use of a different analytical methodology than the one for which the study was designed, may constitute a threat to the internal validity of the results.
  Similar threats may be the lack of control group in both studies, which is in contrast to the recommendation from Ryan and Smith [17], and the lack of information on the degree of implementation of SBABI by each study group/cluster. Nevertheless, the mixed effect model analysis methodology will consider a separate pattern of the relationship between effectiveness and time for each study group/cluster. This may partially compensate for the two previous issues, since study groups/clusters with less effectiveness will be allowed to follow a different pattern as opposed to those with more effectiveness.
  Another threat to the internal validity may be due to the attempt to harmonize the available data between the two studies. The analysis methodology for doing so may impose different assumptions and may result in loss of some information.
  Two additional challenges derive from in one hand the distribution of anonymous questionnaires to pupils and on the other hand the hierarchical structure of school data (i.e. pupils nested within classrooms, within grades and within schools). The latter will be taken into account using mixed models effects statistical methodology for nested data. This approach is also consistent with the recommendation from Ryan and Smith [17]. Regarding the use of anonymous questionnaire data, the unit of analysis will no longer be at the pupil level but at the study group/cluster level.
  Finally, a potential threat to the internal validity may be introduced by the use of some statistical methodologies. The statistical methodologies planned to be used in the secondary analysis are based on several assumptions about the underlying distributions of the data. Deviations from these assumptions may be a threat for the internal validity of the results of *SET*-*Bullying*. In order to address such a vulnerability, where appropriate, the assumptions of the statistical analysis methodologies will be kept to a minimum and any observed deviation from them will be discussed while reporting the results.
6. “*Selection”* [14] of control or intervention study groups [7]. Since none of the studies included a comparison group, this type of vulnerability due to selection and assignment of study groups to intervention or comparison groups is considered minimal. However, for *DFE*-*SHEFFIELD* only four primary schools decided to continue implementing the intervention and performed the third measurement [8]. The selection of these schools was based on their management willingness [8]. In *RESPEKT*, not all study groups had the chance to participate to all study measurements [9].
  For this reason, all data from all study groups in both studies will be considered for analysis, as they can provide information that can inform the statistical models regarding the shape and magnitude of the relationship between time and effectiveness. Therefore, although selection bias will not be avoided, it will be taken into consideration for the collaborative project.
7. Changes in the study groups due to “*participant* [changes or] *attrition*” [14]. As mentioned above not all study groups participated to all measurements. Within each study group the number of pupils participating to each study measurement varied slightly over time. Although there is no reported mechanism of missing data, this kind of bias cannot be excluded. Having used anonymous questionnaires, a common practice in bullying research [7], it is not feasible to identify if the same pupils have responded in all measurements. Therefore, it cannot be excluded that some pupils involved in bullying may have been more prone to absenteeism and therefore missing systematically some study measurements [7]. This threat to internal validity is impacting also the project results.
8. Any “*interactive effect*” [14] of the above mentioned criteria. Any dynamic effect of a combination of the above sources of vulnerability to the internal validity of the results of *SET*-*Bullying* cannot be excluded.

### External validity

In this subsection, we discuss the external validity of the results of *SET*-*Bullying*. Similarly to the internal validity, the external validity of the results from *SET*-*Bullying* depends not only on the external validity of the original studies but also on the external validity of the methodology used in this collaborative project.

The external validity is important as it would allow the utilisation of the methodology and the outcomes of *SET*-*Bullying* in other studies in bullying research. The discussion on the external validity is based on the criteria suggested by Green and Glasgow [15]. As per these criteria, the external validity of *SET*-*Bullying* is influenced by the following factors:

1. The “*reach and representativeness*” [15] of the original studies. The studies that have contributed data were designed and implemented in specific contexts, time periods, geographical regions and on populations. For instance, *RESPEKT* was a pilot study implemented on a smaller population. *DFE*-*SHEFFIELD* was a much larger project, which was implemented in 24 schools, but only 4 of them participated in the follow-up assessment. The external validity of the collaborative project has vulnerabilities originating from the original studies that have contributed data.
  Despite the SBABI differences, the same analysis will be performed for both studies. The statistical modelling approach will be rather generic and independent of the specific characteristics of the SBABIs. Therefore, it would not be unreasonable to assume that the form of the relationship between time and effectiveness of SBABI may hold for all kinds of similar interventions, although its magnitude may vary depending on the characteristics and the effectiveness of each SBABI.
  The analysis will be based on pupils’ self-reported frequencies of been bullied and bullying others. As Chalamandaris and Piette [7] describe, such self-reported frequencies are commonly used in the evaluation of the effectiveness of SBABIs. However, other types of BROs (such as intentions, attitudes, feelings, perceptions) or reports from different sources (such as teachers, parents, other stakeholders, observations, archival records) have also used in other studies, as BROs in concluding on the effectiveness of SBABIs [7]. Therefore, this is a vulnerability of *SET*-*Bullying* since the under investigation relationship will not be evaluated for other types of BROs or information collected from other stakeholders of the school community. Thus, any extrapolation is not considered feasible.
  Additionally, *SET*-*Bullying* included only two studies out of a pool of 22 studies. This pool was based on a literature review [7] which included articles published “*prior to the end of January 2008*”. Given the variability in terms of SBABI characteristics and study design characteristics among these 22 studies, the two participating studies are not representative of the pool of the 22 studies. Therefore, only a small portion of the available SBABI literature is included in *SET*-*Bullying*. This constitutes a threat to the external validity of its results.
2. The “*implementation*” and “*adaptability*” [15] of the statistical modelling process. The degree of “*implementation*” of the original SBABI or their “*adaptability*” in a different context should not bring any vulnerability to *SET*-*Bullying*. The reason is that the statistical modelling suggested by *SET*-*Bullying* will be rather independent of specific characteristics of the SBABI that have contributed data. It is also assumed that the relationship between time and effectiveness of SBABI may stand under various scenarios of effectiveness (i.e. from minimally to rather effective SBABIs). The numerical magnitude of that relationship is expected to differ between these scenarios.
  Additionally, the ability to implement the same statistical models to any other set of effectiveness data from SBABI should be consider possible. In all disseminations of the statistical modelling of *SET*-*Bullying* the statistical methodology will be clearly stated and described giving the possibility of replication in other datasets. For this end, the selection of variables to be included will be rather basic including the time of measurement and two demographic characteristics, i.e. pupils’ age and gender.
3. The types of “*outcomes*” [15] and their potential future use. The outcome of the original studies was to conclude on the effectiveness of SBABI. *SET*-*Bullying* has a different aim which is to describe and predict the relationship between time and effectiveness of SBABI. The analysis methodology is expected to achieve this aim independently of whether the SBABI has been found to be effective or its effectiveness has limited external validity. Therefore, any vulnerability to external validity of the original studies, regarding their outcomes is not directly impacting the external validity of the collaborative project.
  This is mostly threatened by the fact that it is a secondary analysis of data from studies that have been designed and implemented for a different aim. It is likely, that if a study was to be designed for the aims of *SET*-*Bullying*, its design and implementation might have been different than the original studies, aiming to better address its aims. Therefore, any outcome from *SET*-*Bullying* can be considered as hypothesis generating and cannot be conclusive for all current or future SBABIs. In order to confirm such a hypothesis, a specific study needs to be conducted for this aim.
4. The further and future efforts for “*replication*” [15] of the results of *SET*-*Bullying*, which Green and Glasgow [15] refer to as “*maintenance andinstitutionalization*”. It is very important to implement in the future the same statistical modelling methodology in other SBABIs that have already been concluded in order to asses whether the results from this collaborative project would have been replicated. The ideal scenario would have been a prospective study dedicated and sufficiently powered to address these aims.

## Conclusions

All the afore mentioned threats to the internal and external validity may not diminish the fact that since the first SBABIs, *SET*-*Bullying* is the first effort to further understand and statistically model the impact of time on the observed effectiveness of SBABIs as well as to assess the predictability of this impact.

Getting further insights into the primary objective of *SET*-*Bullying* would allow for better planning of SBABIs and for more optimized impact evaluation practices. Furthermore, if the prediction aim of this effort lead up to some concrete results, having prior estimation of the medium-term or long-term effectiveness, may be crucial for health promotion planners. It would allow them to better assign resources and plan long-term health promotion anti-bullying strategies and interventions.

Further perspectives could include the implementation of the same analysis methodology in different BROs and more datasets, than the ones included in this collaborative project. Additionally, the analysis methodology of *SET*-*Bullying* could inspire similar explorations in order to understand the relationship between time and effectiveness of health promotion interventions addressing other health promotion issues, as well as to assess the predictability of their medium-term or long-term effectiveness.
